# Beyond clinical trials: real-world impact of immunotherapy on NSCLC in Jordan

**DOI:** 10.3389/fonc.2024.1369126

**Published:** 2024-04-30

**Authors:** Taher Abu Hejleh, Karim AlSawalha, Sufian Abdel Hafiz, Tamer Al-Batsh, Roaa Abu Hejleh, Sameer Yaser, Husam Abu Jazar, Jamal Khader, Anoud Alnsour, Issa Mohamad, Riad Abdel Jalil, Ahmad Abu-Shanab, Azza Gharaibeh, Mohammad Abu Shattal, Akram Alibraheem, Hussam Haddad, Naser Mahmoud, Shadi Obeidat, Mohammed J. Al-Jaghbeer, Muhammad Furqan, Alessio Cortellini, Vamsidhar Velcheti, Kamal Al-rabi

**Affiliations:** ^1^ Department of Internal Medicine, Medical Oncology, King Hussein Cancer Center, Amman, Jordan; ^2^ Department of Internal Medicine, Hematology, Oncology and Blood and Marrow Transplantation, University of Iowa, Iowa City, IA, United States; ^3^ Department of Radiation Oncology, King Hussein Cancer Center, Amman, Jordan; ^4^ Department of Surgery, Thoracic Surgery, King Hussein Cancer Center, Amman, Jordan; ^5^ Department of Radiology, King Hussein Cancer Center, Amman, Jordan; ^6^ Department of Nuclear Medicine, King Hussein Cancer Center, Amman, Jordan; ^7^ Department of Pathology, King Hussein Cancer Center, Amman, Jordan; ^8^ Department of Internal Medicine, Pulmonary and Critical Care Medicine, King Hussein Cancer Center, Amman, Jordan; ^9^ Operative Research Unit of Medical Oncology, Fondazione Policlinico Universitario Campus Bio-Medico, Rome, Italy; ^10^ Department of Surgery and Cancer, Imperial College London, London, United Kingdom; ^11^ Grossman School of Medicine, New York University, New York, NY, United States; ^12^ School of Medicine, University of Jordan, Amman, Jordan

**Keywords:** Jordan, KHCC, immunotherapy, NSCLC, real-world

## Abstract

**Background:**

This study aims to evaluate real-world (rw) outcomes of immunotherapy (IO) for advanced stage NSCLC at King Hussein Cancer Center (KHCC) in Jordan.

**Methods:**

Advanced stage NSCLC patients who received IO at KHCC between 2017 and 2022 were included. The data were retrospectively collected. PFS and OS were estimated for patients with ECOG performance status (ECOG PS) 0-1. Cox regression analyzed predictors of OS in first-line (1L) IO, regardless of performance status.

**Results:**

The total number of patients included was 244. Out of those, 160 (65%), 67 (28%), and 17 (7%) patients received IO as 1L, second-line (2L), or third-line or beyond (3L or beyond), respectively. The median age for all patients was 59 years. Male were 88%, and 77% were smokers. The median follow-up time was 12.5 months. The median PFS and OS for 1L IO were 7 [95% CI 5.8 – 10.3] and 11.8 [95% CI 8.8 – 14.4], months, respectively. In the first 3 months after starting 1L IO, 34/160 (21%) patients had died. For those who survived beyond 3 months after starting 1L IO, the median PFS and OS were 11.3 [95% CI 8.3 – 16.5] and 15.4 [95% CI 13.2 – 21] months, respectively. In the Cox regression model of 1L IO patients with any performance status, ECOG PS 2 was predictive of worse OS compared to ECOG PS 0-1 (*p*= 0.005).

**Conclusion:**

This real-world study of advanced-stage NSCLC patients treated with immunotherapy at KHCC reveals outcomes that fall short of those anticipated from clinical trials. The inclusion of Middle Eastern patients in lung cancer trials is essential to ensure adequate representation of various ethnicities in clinical research.

## Introduction

Lung cancer ranks as the fourth most common cancer in Jordan, comprising 6.5% of all annual cancer cases. In males, it is the second most prevalent type of cancer and the primary cause of cancer-related mortality, accounting for 22.4% of all cancer deaths among men (1). The smoking prevalence in Jordan reaches up to 51% (2) and the average age for lung cancer diagnosis was reported as 63.8 years (3).

Immunotherapy (IO) has revolutionized the treatment of advanced-stage non-small cell lung cancer (NSCLC). Utilizing the patient’s immune system to combat cancer, IO has demonstrated significant efficacy in these patients. The advent of checkpoint inhibitors, including PD-1 and PD-L1 inhibitors, has enhanced clinical outcomes, yielding improved tumor response rates and extending survival compared to conventional cytotoxic chemotherapy (4). The 5 year overall survival estimate for stage IV NSCLC patients with PD-L1 expression of ≥50% who received single agent pembrolizumab, a PD-1 inhibitor, was 32% (5).

Clinical trials have underscored the importance of PD-L1 immunohistochemistry as a crucial biomarker for assessing the effectiveness of immunotherapy (IO) in treating NSCLC (6). This finding emerged from extensive research on single-agent IO (4), IO-chemotherapy combinations (7, 8), and IO-IO combinations (9). Consequently, evaluating PD-L1 expression levels in advanced-stage NSCLC has become a critical aspect of treatment planning. The PD-L1 expression in a lung cancer patient population from the Middle East was found to be similar to published literature (10).

The objective of this study is to delineate the clinical characteristics and treatment outcomes of advanced-stage NSCLC patients treated with IO at King Hussein Cancer Center (KHCC) in Jordan, particularly since the introduction of pembrolizumab at KHCC in 2017. This research holds significant global implications, as it addresses a notable gap in survival outcomes data for NSCLC patients receiving immunotherapy in the Middle East, a region often underrepresented in international clinical trials (11). The findings of this study contribute to a better understanding of the outcomes of IO across diverse patient populations.

## Materials and methods

### Study design and patient population

This retrospective study investigates the outcomes of advanced-stage NSCLC patients with a favorable performance status (ECOG PS 0-1) who underwent immunotherapy (IO) at King Hussein Cancer Center (KHCC) in Jordan. Throughout the study, data confidentiality and privacy were rigorously upheld in line with the Health Insurance Portability and Accountability Act (HIPAA) standards. Adherence to the ethical principles outlined in the Declaration of Helsinki was ensured. The study protocol received the requisite approval from the Institutional Review Board (IRB) at KHCC (IRB number: 23 KHCC 020), and the IRB granted a waiver for informed consent.

The study population included patients ≥18 years old diagnosed with advanced stage NSCLC who had received at least one IO treatment between December 2017 and February 2022. For analyses, only patients with ECOG PS 0-1 were included, except for the univariate and multivariate analysis where patients with any performance status (ECOG PS 0-4) were included. The total number of patients with ECOG PS 2-4 was 67. Excluding patients with poor ECOG PS from the PFS and OS survival analysis aimed to ensure a cohort resembling NSCLC patients in clinical trials, facilitating more meaningful comparisons of survival figures. Then, ECOG PS 2-4 cases were added to the univariate and multivariate analysis to identify the impact of poor ECOG PS, among other variables, on survival specifically in our patient population. The first approved IO at KHCC was pembrolizumab. Patients received IO as monotherapy or with chemotherapy. Demographic and clinical characteristics were categorized by IO line of treatment (1L, 2L, 3L or beyond).

### Data sources

A structured database was built from patient information in the VISTA CPRS Electronic Medical Record (EMR) using Fileman. Data cleaning and analysis were performed with Microsoft Excel, Python, and R. To ensure accuracy, a random sample underwent manual verification by cross-checking with original VISTA CPRS EMR records to identify discrepancies or errors.

### Study variables and outcome measures

We gathered patient data, including demographics, clinical features, and treatment specifics. Key time points, such as diagnosis dates, treatment initiation, post-immunotherapy (IO) progression, last known alive status, and death dates, were recorded for outcome assessment. To prevent biases, we used the date of IO initiation, not the diagnosis date, as the reference point for survival analysis.

The study’s primary endpoints were progression-free survival (PFS) and overall survival (OS). For OS, the defining event was death. In contrast, for PFS, the event was identified as either the date of radiographic progression as determined by the official radiology read, the date of starting subsequent line of treatment following IO, or death, whichever occurred first. The most recent survival date was established based on the latest of several criteria: the last known inpatient admission, the most recent emergency room visit, the date of the last recorded vital signs, or the date of the last clinic visit.

### Statistical analysis

Descriptive statistical methods were employed to evaluate baseline characteristics, stratified by the line of immunotherapy (IO) treatment. We presented categorical data as frequencies and continuous variables as medians with their respective ranges. To discern significant differences between groups, we applied Wilcoxon Rank Sum tests for continuous variables, Pearson’s Chi-Squared tests, and Fisher’s Exact tests for categorical data. In our survival analysis, the Kaplan-Meier method was utilized to estimate overall survival (OS) and progression-free survival (PFS), employing log-rank tests to determine statistical significance. Additionally, a Cox proportional hazards model was employed in the multivariate analysis to evaluate the effects of various covariates on survival outcomes, with results reported as hazard ratios (HRs) and 95% confidence intervals (CIs). All statistical analyses were conducted using R software, version 4.2.2.

## Results

### Patients and treatments

Of 244 patients with advanced stage NSCLC, 160 (65%), 67 (28%), and 17 (7%) received 1L, 2L, or 3L or beyond IO, respectively. Patients with known *EGFR* or *ALK* alterations were excluded. Patients and disease characteristics are listed in **Table 1**. The median age for all patients was 59 (range: 26 -86) years. Patients who received 3L or beyond were more likely to have younger age (median age was 60 years in 1L, 58 years in 2L, and 51 years in 3L or beyond, *p* = 0.004). Most of the patients were men (87%), and were smokers (75%) or ex-smokers (13%). All patients included had ECOG PS of 0-1 (100%). Numerically, more patients received 3L or beyond IO who had stage III NSCLC upon initial diagnosis compared to 1L and 2L patients (29% vs 24% and 7.5%, *p* = 0.054) and more who had received CCRT (29% vs 21% and 4.5%, *p* < 0.002), respectively. There was no significant difference between the small numbers of patients who received neoadjuvant or adjuvant chemotherapy upon initial diagnosis of NSCLC. The most common NSCLC histology was non-squamous (67%). The patients who received 1L IO were more likely to have a PD-L1 ≥50% (*p* < 0.001) and to receive IO and chemotherapy combination (*p* < 0.001). All patients received pembrolizumab except for 1 patient who received nivolumab.

**Table 1:** Demographic and clinical characteristics of advanced stage NSCLC patients (ECOG PS 0 -1) who received first line, second line, or third line or beyond IO.

Demographics and clinical characteristics	All patients, N = 244	IO line of treatment			
		1L IO, N = 160 (%)	2L IO, N = 67 (%)	3L or beyond IO, N = 17 (%)	p-value*^1^*
Age at diagnosis					0.004
Median (Range)	59 (26 -86)	60 (33 - 86)	58 (26 - 80)	51 (33 - 72)	
Gender					0.4
Male	213 (87%)	141 (88%)	59 (88%)	13 (76%)	
Female	31 (13%)	19 (12%)	8 (12%)	4 (24%)	
Smoking History					0.6
Never-smoker	29 (12%)	17 (11%)	8 (12%)	4 (24%)	
Ex-smoker	32 (13%)	20 (12%)	10 (15%)	2 (12%)	
Smoker	183 (75%)	123 (77%)	49 (73%)	11 (65%)	
ECOG Performance Status					
0-1	244 (100%)	160 (100%)	67 (100%)	17 (100%)	
Cancer stage at initial diagnosis					0.054
I	4 (1.6%)	2 (1.3%)	2 (3.0%)	0 (0%)	
II	6 (2.5%)	4 (2.5%)	2 (3.0%)	0 (0%)	
III	48 (20%)	38 (24%)	5 (7.5%)	5 (29%)	
IV	186 (76%)	116 (72%)	58 (87%)	12 (71%)	
Neoadjuvant chemotherapy					0.7
Yes	9 (3.7%)	6 (3.8%)	2 (3.0%)	1 (5.9%)	
No	235 (96%)	154 (96%)	65 (97%)	16 (94%)	
Adjuvant chemotherapy					>0.9
Yes	10 (4.1%)	7 (4.4%)	3 (4.5%)	0 (0%)	
No	234 (96%)	153 (96%)	64 (96%)	17 (100%)	
Concurrent chemotherapy and radiation therapy					0.002
Yes	41 (17%)	33 (21%)	3 (4.5%)	5 (29%)	
No	203 (83%)	127 (79%)	64 (96%)	12 (71%)	
Histology					0.2
Non-squamous	164 (67%)	112 (70%)	39 (58%)	13 (76%)	
Squamous	80 (33%)	48 (30%)	28 (42%)	4 (24%)	
PD-L1 TPS (22C3)					<0.001
<1%	1 (0.4%)	0 (0%)	1 (1.5%)	0 (0%)	
1 - <50%	112 (46%)	59 (37%)	43 (64%)	10 (59%)	
≥50%	131 (54%)	101 (63%)	23 (34%)	7 (41%)	
IO used					0.3
Pembrolizumab	243 (100%)	160 (100%)	66 (99%)	17 (100%)	
Nivolumab	1 (0.4%)	0 (0%)	1 (1.5%)	0 (0%)	
IO monotherapy vs. with chemotherapy combination					<0.001
IO monotherapy	185 (76%)	102 (64%)	66 (99%)	17 (100%)	
IO with chemotherapy combination	59 (24%)	58 (36%)	1 (1.5%)	0 (0%)	
Number of IO cycles					0.001
Median (Range)	6 (1 - 43)	8 (1 - 43)	4 (1 - 35)	6 (2 - 32)	
Systemic treatment subsequent to progression on IO					0.8
Yes	68 (28%)	47 (29%)	17 (25%)	4 (24%)	
No subsequent treatment or unknown	176 (72%)	113 (71%)	50 (75%)	13 (76%)	
Type of systemic treatment subsequent to progression on IO^2^					0.049
Platinum based	23 (34%)	20 (43%)	2 (12%)	1 (25%)	
Non-platinum based	45 (66%	27 (57%)	15 (88%)	3 (75%)	

^1^ Kruskal-Wallis rank sum test; Fisher’s exact test; Pearson’s Chi-squared test.

^2^ The denominator in this variable’s percentage is the number of patients who received systemic treatment subsequent to progression on IO.

The median number of IO cycles received was 6 (range, 1-43) cycles. The patients who received 1L IO had significantly higher median number of IO treatments, 8 (range 1-43) cycles compared to 2L IO and 3L or beyond IO patients who received 4(range 1-35) and 6(range 2-32) cycles, respectively (*p* = 0.001). Around one quarter (28%) of all patients who were treated with IO received subsequent systemic treatment upon progression on IO. Around half of the 1L IO patients (43%) received platinum based chemotherapy upon progression on IO.

### Sequential introduction of IO into the advanced stage NSCLC treatment lines at KHCC

KHCC added IO for treating advanced stage NSCLC at different times. Pembrolizumab was approved on December 13, 2017, as 1L treatment if PD-L1 was ≥ 50%, and on January 10, 2018, as 2L treatment if PD-L1 was ≥ 1%. Approval was expanded on November 10, 2019, for non-squamous NSCLC with PD-L1 ≥1%, combining IO and chemotherapy as 1L treatment. On January 25, 2023, advanced squamous NSCLC with PD-L1 ≥1% became eligible for pembrolizumab and chemotherapy. This staggered introduction explains differences in 1L IO patients, more likely to have PD-L1 ≥50% (p < 0.001) and receive IO and chemotherapy combination (p < 0.001). Formulary decisions at KHCC are guided by cost-effectiveness analyses.

**Figure 1:**
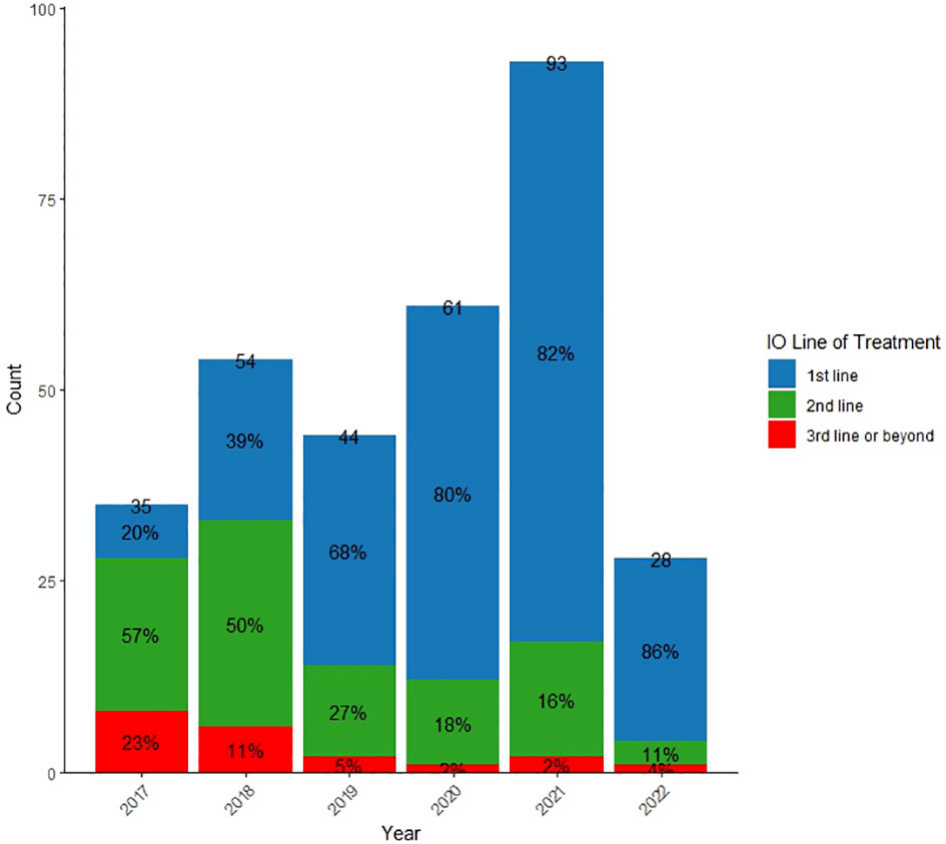
Distribution of IO line of treatment by year of starting IO.

**Figure 1** shows the evolving usage of IO in the treatment of NSCLC at KHCC from 2017 to 2022. The unique NSCLC patients receiving IO at KHCC increased from 44 in 2019 to 93 in 2021. In the 1L setting, there was a consistent rise, reaching 82% in 2021, compared to 39% in 2018. Conversely, the use of IO as 2L treatment decreased from 50% in 2018 to 16% in 2021, and only 2% of patients received IO as 3L or beyond in 2021.

### Survival outcomes

#### Survival for patients after 1L, 2L, and 3L or beyond IO

**Figure 2:**
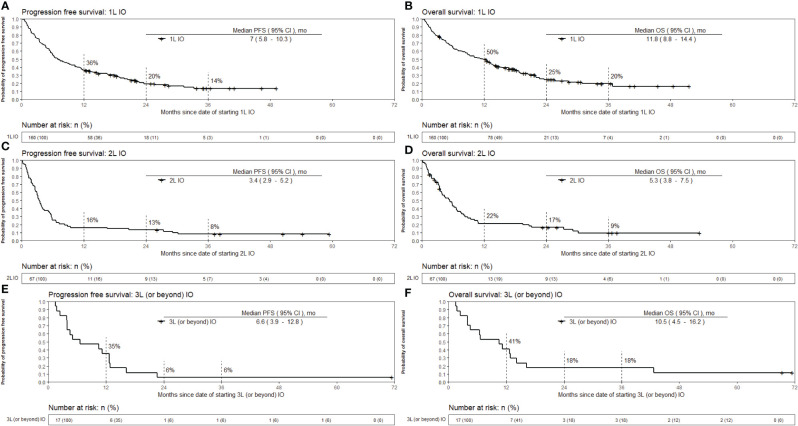
Kaplan-Meier estimates of overall survival and progression free survival from the date of starting immunotherapy. The patients with EGFR mutation in exon 19 or 21, ALK positivity by IHC or rearrangement by FISH, or ROS1 rearrangement by FISH were excluded from the survival analysis. **(A)** OS for 1L IO **(B)** OS for 2L IO **(C)** OS for 3L or beyond IO **(D)** PFS for 1L IO **(E)** PFS for 2L IO **(F)** PFS for 3L or beyond IO. OS, Overall survival; PFS, Progression free survival; IO, Immunotherapy; 1L, First line; 2L, Second line; 3L, Third line; mo, months.

The median follow up time for all patients was 12.1 months. The median follow up time for 1L, 2L and 3L or beyond IO was 12.6, 5.8, and 10.9 months, respectively. Progression-free survival (PFS) and overall survival (OS) for 1L, 2L and 3L or beyond IO are shown in **Figure 2**. Median PFS was the longest for patients who received 1L IO, 7 [95% CI 5.8 – 10.3] months. The median PFS for 2L and 3L or beyond IO was 3.4 [95% CI 2.9 – 5.2] months and 6.6 [95% CI 3.9 – 12.8] months, respectively. Median OS was the longest for patients who received 1L IO, which was 11.8 [95% CI 8.8 – 14.4] months. Median OS for 2L and 3L or beyond IO was 5.3 [95% CI 3.8 – 7.5] months and 10.5 [95% CI 4.5 – 16.2] months, respectively. Survival for 1L, 2L and 3L or beyond IO at 36 months since initiation of IO was 20%, 9%, and 18%, respectively.

#### Survival for patients who received 1L IO and survived ≥ 3 months after starting IO

**Figure 3:**
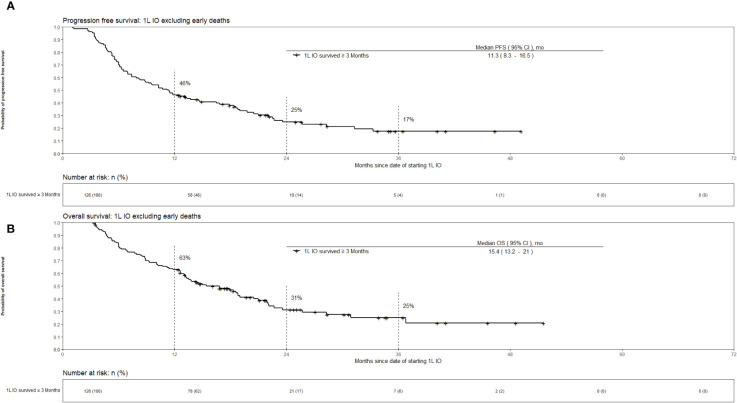
Kaplan-Meier estimates of overall survival for advanced stage NSCLC who received 1L IO excluding early deaths (survival <3 months). Part **(A)** progression free survival. Part **(B)** overall survival.

Out of the 160 patients who received 1L IO, 34 (21%) died within 3 months of initiating IO. The survival outcomes for the patients who received 1L IO and survived 3 months or more after starting IO are shown in **Figure 3**. The median PFS was 11.3 [95% CI 8.3 – 16.5] months, and the median OS was 15.4 [95% CI 13.2 – 21] months. Survival at 36 months was 25%. **Table 2** shows the characteristics of the patients who received 1L IO and survived <3 versus ≥ 3 months. Those who survived ≥ 3 months were more likely to receive subsequent systemic treatment after progression on IO (*p* < 0.001). The median number of IO cycles administered for the patients who survived ≥ 3 months was 11 (range 1-43).

**Table 2:** Characteristics of advanced stage NSCLC patients who received first line according to survival time (<3 vs ≥3, months).

Demographics and clinical characteristics	Survival Time		p-value*^1^*
	<3 months (Early Death), N = 34(%)	≥3 months, N = 126(%)	
Age at diagnosis			0.4
Median (Range)	60 (39 - 77)	60 (33 - 86)	
Gender			0.9
Male	30 (88%)	111 (88%)	
Female	4 (12%)	15 (12%)	
Smoking History			>0.8
Never-smoker	4 (12%)	13 (10%)	
Ex-smoker	3 (8.8%)	17 (13%)	
Smoker	27 (79%)	96 (76%)	
ECOG Performance Status			
0-1	34 (100%)	126 (100%)	
Cancer stage at initial diagnosis			>0.9
I	0 (0%)	2 (1.6%)	
II	1 (2.9%)	3 (2.4%)	
III	9 (26%)	29 (23%)	
IV	24 (71%)	92 (73%)	
Neoadjuvant chemotherapy			0.6
Yes	2 (5.9%)	4 (3.2%)	
No	32 (94%)	122 (97%)	
Adjuvant chemotherapy			0.2
Yes	3 (8.8%)	4 (3.2%)	
No	31 (91%)	122 (97%)	
Concurrent chemotherapy and radiation therapy			0.6
Yes	8 (24%)	25 (20%)	
No	26 (76%)	101 (80%)	
Histology			0.7
Non-squamous	23 (68%)	89 (71%)	
Squamous	11 (32%)	37 (29%)	
PD-L1			0.5
<1%	0 (0%)	0 (0%)	
1 - <50%	11 (32%)	48 (38%)	
≥50%	23 (68%)	78 (62%)	
IO used			>0.9
Pembrolizumab	34 (100%)	126 (100%)	
Nivolumab	0 (0%)	0 (0%)	
IO monotherapy vs. with chemotherapy combination			0.2
IO monotherapy	25 (74%)	77 (61%)	
IO with chemotherapy combination	9 (26%)	49 (39%)	
Number of IO cycles			<0.001
Median (Range)	2 (1 - 3)	11 (1 - 43)	
Systemic treatment subsequent to progression on IO			<0.001
Yes	1 (2.9%)	46 (37%)	
No subsequent treatment or unknown	33 (97%)	80 (63%)	
Type of systemic treatment subsequent to progression on IO^2^			0.4
Platinum based	1 (100%)	19 (41%)	
Non-platinum based	0 (0%)	27 (59%)	

^1^ Wilcoxon rank sum test; Pearson’s Chi-squared test; Fisher’s exact test.

^2^ The denominator in this variable’s percentage is the number of patients who received systemic treatment subsequent to progression on IO.

#### Survival of patients based on receiving IO monotherapy versus IO in combination with chemotherapy, and the PD-L1 status

Survival for 1L IO patients categorized by IO monotherapy versus IO with chemotherapy combination, and PD-L1 expression were also explored. The patients who received IO in combination with chemotherapy had better overall survival compared to those who received IO monotherapy in the 1L setting; however, the difference in survival was not statistically significant [HR 0.81 (95% CI 0.55 – 1.2), *p* = 0.3]. Regarding PD-L1 expression, the patients who received 1L IO had better survival in the first 12 months after starting IO if PD-L1 was 1 - < 50% compared to ≥50%; however, the overall survival over the follow up period for the study was not significantly different based on the PD-L1 status [HR 1.07 (95% CI 0.73 – 1.56), *p* = 0.74]. There was no significant overall survival difference for the 2L patients based on PD-L1 level of expression [HR 1.13 (95% CI 0.69 – 1.84), *p* = 0.64]. However, the 3L or beyond IO patients who had PD-L1 expression ≥50% survived longer compared to those with PD-L1 of 1 - < 50% [HR 0.36 (95% CI 0.13 – 1.01), *p* = 0.05].

### Cox Proportional hazard model for predictors of overall survival

The univariate and multivariate Cox Proportional Hazard models for predictors of overall survival are presented in **Table 3**. These hazard models included patients who received 1L only and had ECOG PS 0-4. The total number of patients included was 207. The only variable that was predictive of overall survival in both the univariate and multivariate analyses was the ECOG PS. Compared to the reference ECOG PS of 0-1, those with ECOG PS 2-4 had worse overall survival on multivariate analysis. [HR 1.83 (95% CI 1.24 – 2.7), *p =* 0.002].

**Table 3:** Univariate and multivariate analysis of overall survival for advanced stage NSCLC patients who received first line IO.

Variable	Univariate			Multivariate		
	HR	95% CI	P	HR	95% CI	P
Age at diagnosis	1.01	1 - 1.03	0.06	1.01	1 - 1.03	0.156
Gender - Male	0.83	0.52 - 1.33	0.45	0.82	0.49 - 1.4	0.476
Smoking History - Never smoker	1.08	0.57 - 2.06	0.8	1.09	0.55 - 2.16	0.813
Smoking History - Smoker	0.96	0.6 - 1.53	0.86	1	0.62 - 1.62	1
ECOG performance status = 2-4	1.86	1.29 - 2.68	0.001	1.83	1.24 - 2.7	0.002
Cancer stage at initial diagnosis - stage II	2.55	0.26 - 24.65	0.42	3.07	0.3 - 31.08	0.342
Cancer stage at initial diagnosis - stage III	1.84	0.25 - 13.56	0.55	1.92	0.24 - 15.65	0.541
Cancer stage at initial diagnosis - stage IV	2.06	0.29 - 14.75	0.47	1.83	0.25 - 13.58	0.553
IO and chemotherapy combination	0.82	0.58 - 1.16	0.26	0.84	0.53 - 1.34	0.458
Received neoadjuvant chemotherapy	1.13	0.46 - 2.75	0.8	1.27	0.48 - 3.37	0.629
Received adjuvant chemotherapy	1.66	0.73 - 3.77	0.23	2.03	0.84 - 4.92	0.116
Received concurrent chemotherapy and radiation therapy	0.82	0.54 - 1.26	0.37	0.76	0.37 - 1.56	0.452
Histology - Squamous	1.03	0.73 - 1.44	0.87	0.93	0.64 - 1.37	0.725
PD_L1≥50%	1.08	0.77 - 1.51	0.66	0.96	0.61 - 1.52	0.868

## Discussion

This rw study from KHCC-Jordan provides a critical examination of IO outcomes in advanced stage NSCLC in a patient population that is under-represented in clinical trials. In the advanced stage NSCLC patients with good performance status who received 1L IO at KHCC, our findings reveal a median OS of 11.8 months, extending to 15.4 months when excluding those who survived less than three months post IO initiation. These figures fall short of the anticipated outcomes from randomized NSCLC IO clinical trials (4, 7, 8), underscoring the necessity of inclusive and diverse patient representation in global lung cancer studies.

Real-world data on outcomes of IO as a 1L treatment of advanced stage NSCLC has been reported in several publications (12–15). Khozin et al. (12) utilized Flatiron Health to study a total of 5257 NSCLC patients who received IO. The median OS for those who received 1L IO was 10.8 months (12). Another study by Velcheti et al. which included patients who received 1L IO, survived at least 6 months and had an ECOG PS of 0-1 showed a median OS of 19.6 months (16). Another retrospective study showed the survival was better for the NSCLC patients who were alive at 12 weeks after starting single agent pembrolizumab compared to the whole cohort; however, the median OS was not reached when the data was published (17). The frequency of death at 12 weeks was 11% for those who received single agent pembrolizumab and 15.2% for the pembrolizumab and chemotherapy combination group (17). In our study, 21% of the patients died within 3 months of starting IO. Deaths in the first 3 months after enrollment in four of the major 1L IO clinical trials in NSCLC were less than what we report in our study (4, 7, 8, 18). This observed early mortality rate after initiation of IO further accentuates the need for prudent patient selection to optimize treatment outcomes and minimize early mortality risks. In **Tables 4**–**6**, we summarize survival outcomes from rw data studies including figures from data from the Middle East.

**Table 4:** First line rw studies.

Author year	Design	Study	number of patients	country	Age	PS ≥2	rwOS (months)	rwPFS (months)	ref
E. Pons−Tostivint 2022	Observational, Multicenter	First line: IO vs IO-CT in more than 50%	141	France	68	22%	12-month OS rate 70.2%	11.3	(17)
Marija Ivanović 2021	Observational, single center	First line: pembrolizumab second-line: Atezolizumab, Nivolumab, or Pembrolizumab	66	Slovenia	64	6%	The 1-year (OS) 62%. Second line 9.9	First line 9.3 Second line 3.5	(15)
David Waterhouse 2021	Observational, Multicenter	First line: I-O plus chemotherapy,	4271	United States	69.0	27%	Squamous 11.3 Non-squamous 14.1	Not reported	(19)
Stephen V. Liu 2022	Observational, Multicenter	First line: I-O plus CT PS: 0-1	377	United States	66	0%	17.2	6.2	(20)
Vamsidhar Velcheti 2019	Observational, Multicenter	First line PD-L1 > 50% Had follow up ≥6 months PS: 0-1	432	United States	72	0%	18.9	6.8	(14)
Christine M. Cramer−van der Welle1 2021	Observational, Multicenter	First IO second line and beyond	83 (first line) 200 (subsequent treatment)	Netherlands	66	4%	15.8	8.9	(21)
Maurice Perol 2022	Observational, Multicenter	First line	521	United States	N/A	0%	22.1	11.5	(22)
Beung-Chul Ahn 2019	Observational, single	All lines Pembrolizumab and Nivolumab	155	Korea	64	22%	10.25	3.06	(23)
Renaud Descourt 2022	Observational, Multicenter	First line Pembrolizumab PD-L1 > 50%	845	France	65	22.2%	29.5	9.2	(24)

N/A, not available.

**Table 5:** Second line rw studies.

Author Year	Type	Country	Study	Number of patients	PS ≥2	number of lines	Median age	Median PFS	Median OS	ref
Martin 2020	Observational Multicenter	Argentine	Nivolumab Second line	109	17(15.6%)	2(1-4)	65	10.2	12.3	(25)
Areses Manrique 2018	observational multicenter	Spain	Nivolumab Second line	188	19(10%)	71 (38%) received 2 or more lines	58	4.8	12.8	(26)
Morita 2019	observational multicenter	Japan	Nivolumab Second line	901	157(17.4%)	2 (1-12)	67	2.1	14.6	(27)
Dudnik 2018	observational multicenter	Israel	Nivolumab Second line	260	119(46%)	68(26%) received 2 or more lines	67	2.8	5.9	(28)
Park 2021	observational multicenter	Korea	pembrolizumab or nivolumab after failure of platinum-based chemotherapy	1181	141(13%)	484 (41%) received 2 or more lines	67	2.9	10.7	(29)
Kobayashi 2018	observational multicenter	Japan	Nivolumab as subsequent line	142	23(16.2%)	85 (60%)	67	58 days	ND	(30)

ND, Not determined.

**Table 6:** rw studies from the Middle East.

Author year	Type of publication	Country	Study	Number of patients	Results	ref
Naser 2022	Abstract	Lebanon	First line	135	Median PFS 1 was 7.7, 14, 11.1 months for chemotherapy, immunotherapy and chemo-immunotherapy respectively (p = 0.062). Median OS was 22.9 months (CI 95%, 17.7-28.1) with no significant correlation with treatment type (p = 0.85)	(31)
Karak 2019	Research article	Lebanon	Second line and beyond	110	Median progression-free survival was 4 months and median overall survival was 8.1 months	(32)
Al Nuhait 2021	Research article	Kingdom of Saudi Arabia	Real-world safety experience with IO in Saudi Arabia	53 (17 patient with lung cancer)	Patients treated with immune checkpoint inhibitors could have a variety of adverse drug events that might lead to treatment discontinuation and increase overall emergency room visits. This study highlights the most common adverse drug events associated with ICIs use at a tertiary care center in Saudi Arabia	(33)
Jazieh 2022	Research article	United Arab Emirates KSA, Kuwait, Egypt, Turkey	Real-world Treatment Patterns and Outcomes in Stage III Non-small Cell Lung Cancer: Middle East and Africa - KINDLE Study	33 centers 1,046	The data reveal an unmet need in stage III NSCLC with worse PFS and OS in the MEA subset than in the global cohort. Better access to newer therapies and quality care will be crucial in improving patient outcomes in the MEA.	(34)
Dudnik 2018	Research article	Israel	Nivolumab Second line	260	Median PFS 2.8 Median OS 5.9 months	(28)

The influence of ECOG PS on treatment outcomes is a significant finding in our study. Patients with ECOG PS 0-1 showed better survival outcomes compared to those with higher PS scores [HR 1.83 (95% CI 1.24 – 2.7), echoing the global literature’s emphasis on the importance of comprehensive patient evaluation before IO initiation. Studies showed that careful consideration is needed when using IO in NSCLC patients with poor PS, higher comorbidity score, and older age (35–37). In a metanalysis of Pembrolizumab as monotherapy or in combination with chemotherapy of 11 randomized clinical trials and 26 retrospective real-world (rw) studies, multivariate analysis showed that an ECOG PS of 0-1 was an independent predictor of longer OS (38).

In our patient population, PD-L1 expression in the 1L IO treatment setting showed an unusual pattern: those with PD-L1 expression 1 - < 50% exhibited improved survival within the initial 12 months of receiving IO compared to those with PD-L1 expression of ≥50%. However, this survival advantage diminished over subsequent follow-up periods. This finding contrasts with the prevailing literature, which typically associates higher PD-L1 expression with better survival outcomes (18). While the exact cause of this observation remains unclear, it’s important to note that the limited sample size may hinder definitive conclusions. Nonetheless, one plausible explanation could be linked to heavy smoking habits, prevalent in Jordan, potentially contributing to elevated PD-L1 expression levels (39) and more smoking-related comorbidities, possibly diluting the benefits of IO among patients with high PD-L1 expression.

The benefit of IO in the 2L setting and beyond in rw data is also of interest. In our study, the patients who received 2L IO had a median PFS and OS of 3.4 [95% CI 2.9 – 5.2] months and 5.3 [95% CI 3.8 – 7.5] months, respectively. In the 2L NSCLC treatment trial, KEYNOTE-010, pembrolizumab showed a survival benefit over docetaxel with a median PFS and OS of 3·9 and 10.4 months, respectively (40). In our study, when compared to KEYNOTE-010, the PD-L1 expression was ≥50% in 34% of the cases who received 2L IO, as opposed to 42% in the latter. In rw data, Khozin et al. reported on the survival of patients who received 2L pembrolizumab showing a median PFS and OS of 3.7 (2.9-4.1) months and 12.0 (9.3-14.7) months, respectively (12). Juergens et al. also published the rw data on using nivolumab as 2L IO in Canada showing a median PFS and OS of 3.5 and 12 months, respectively (41). In a metanalysis that included 32 rw studies of 2L IO in NSCLC, safety and efficacy of IO in the rw data were comparable to the figures from randomized clinical trials with a median PFS and OS of 3.35 months and 9.98 months, respectively (42). Although the PFS in our data is similar to the published figures in clinical trials and rw data, the lower OS in our study could be due to comorbid conditions that were not captured in our study. Regarding the patients who received 3L or beyond IO, our study had a total of 17 patients. Their median PFS and OS since initiating IO were 6.6 and 10.5 months, respectively. Higher PD-L1 expression was associated with longer OS in this subgroup of patients. However, we need to exercise caution when interpreting survival figures for these patients given the small numbers and possibly selection bias of the healthiest to receive more cancer treatments.

Our study brings into focus the critical gap in the representation of Middle Eastern patients in global clinical trials. Ethnic and genetic diversity can significantly influence the efficacy and safety profile of IO therapies. Pharmacogenomics variations, lifestyle and environmental factors, prevalent comorbidities, and healthcare access disparities can modulate treatment responses (43, 44). In Jordan, the high prevalence of smoking, particularly among males (2), and the younger age at lung cancer diagnosis may result in distinct disease profiles, influencing treatment outcomes. Our study showed that 87% of the patients were males, 88% were smokers or ex-smokers, and the median age of lung cancer diagnosis was 59. Therefore, including more ethnically diverse populations in clinical trials is essential to ensure that the findings are reflective of a global patient population.

Limitations of our study include the retrospective nature of the data, which comes from a single cancer center in the Middle East and the relatively short median follow up time of 12.1 months. The authors recognize the importance of a larger sample size that includes a more diverse representation of Middle Eastern countries to improve generalizability of the findings to the region. This is especially important because different Middle Eastern countries have different ethnicities, healthcare practices and access to IO.

In conclusion, our study showed shorter survival in advanced stage NSCLC patients with good PS receiving 1L IO compared to randomized clinical trials. Even after excluding early deaths (21% of patients), survival remained suboptimal. This study serves as a clarion call for more inclusive and diverse clinical research, advocating for further participation of patients from the Middle East and other parts of the world. It stresses the importance of careful patient selection for IO therapy in NSCLC and underscores the need to account for ethnic diversity to enhance the generalizability and applicability of clinical trial outcomes. Our real-world research, alongside others, invites the establishment of real-world benchmarks for IO outcomes, complementing the outcomes of clinical trials. Such benchmarks could offer valuable insights for researchers and regulatory authorities evaluating the effectiveness of IO in lung cancer across diverse global populations.

## Data availability statement

The raw data supporting the conclusions of this article will be made available by the authors, without undue reservation.

## Ethics statement

The studies involving humans were approved by King Hussein Cancer Center, IRB No: 23 KHCC 20, IRB Approval Date: February 26, 2023. The studies were conducted in accordance with the local legislation and institutional requirements. Written informed consent for participation was not required from the participants or the participants’ legal guardians/next of kin in accordance with the national legislation and institutional requirements.

## Author contributions

TA: Conceptualization, Data curation, Formal analysis, Funding acquisition, Investigation, Methodology, Project administration, Resources, Software, Supervision, Validation, Visualization, Writing – original draft, Writing – review & editing. KA: Data curation, Writing – original draft, Writing – review & editing, Methodology, Software, Validation. RAH: Validation, Writing – review & editing, Data curation. SA: Data curation, Methodology, Writing – review & editing. TA-B: Validation, Writing – review & editing. SY: Writing – review & editing. HA: Writing – review & editing. JK: Writing – review & editing. AA: Writing – review & editing. IM: Writing – review & editing. RAJ: Writing – review & editing. AAS: Writing – review & editing. AG: Writing – review & editing. MA: Writing – review & editing. AA-I: Writing – review & editing. HH: Writing – review & editing. NM: Writing – review & editing. SO: Writing – review & editing. MA-J: Writing – review & editing. MF: Writing – review & editing. AC: Writing – review & editing. VV: Writing – review & editing. KA-R: Validation, Writing – original draft, Writing – review & editing.

## References

[1] Jordan cancer registry cancer incidence in Jordan. (2018) 72(6):1881–90.

[2] Alkouri O Khader Y Al-Bashaireh AM. Prevalence of cigarettes and waterpipe smoking among Jordanians, refugees, and migrants in Jordan and its associated factors: A secondary data analysis. Int J Environ Res Public Health. (2022) 20(1):82. doi: 10.3390/ijerph20010082 36612400 PMC9819960

[3] Alqudah MA Alfaqih MA Hamouri S Al-Shaikh AF Haddad HK Al-Quran WY. Epidemiology and histopathological classification of lung cancer: A study from Jordan, retrospective observational study. Ann Med Surg (Lond). (2021) 65:102330. doi: 10.1016/j.amsu.2021.102330 33996061 PMC8094892

[4] Reck M Rodríguez-Abreu D Robinson AG Hui R Csőszi T Fülöp A. Pembrolizumab versus chemotherapy for PD-L1-positive non-small-cell lung cancer. N Engl J Med. (2016) 375:1823–33. doi: 10.1056/NEJMoa1606774 27718847

[5] Reck M Rodríguez-Abreu D Robinson AG Hui R Csőszi T Fülöp. Five-year outcomes with pembrolizumab versus chemotherapy for metastatic non–small-cell lung cancer with PD-L1 tumor proportion score ≥ 50%. J Clin Oncol. (2021) 39:2339–49. doi: 10.1200/jco.21.00174 PMC8280089 33872070

[6] Memmott RM Wolfe AR Carbone DP Williams TM. Predictors of response, progression-free survival, and overall survival in patients with lung cancer treated with immune checkpoint inhibitors. J Thorac Oncol. (2021) 16:1086–98. doi: 10.1016/j.jtho.2021.03.017 33845212

[7] Paz-Ares L Luft A Vicente D Tafreshi A Gümüş M Mazières J. Pembrolizumab plus chemotherapy for squamous non-small-cell lung cancer. N Engl J Med. (2018) 379:2040–51. doi: 10.1056/NEJMoa1810865 30280635

[8] Gandhi L Rodríguez-Abreu D Gadgeel S Esteban E Felip E De Angelis F. Pembrolizumab plus chemotherapy in metastatic non-small-cell lung cancer. N Engl J Med. (2018) 378:2078–92. doi: 10.1056/NEJMoa1801005 29658856

[9] Paz-Ares L Ciuleanu TE Cobo M Schenker M Zurawski B Menezes J. First-line nivolumab plus ipilimumab combined with two cycles of chemotherapy in patients with non-small-cell lung cancer (CheckMate 9LA): an international, randomised, open-label, phase 3 trial. Lancet Oncol. (2021) 22:198–211. doi: 10.1016/s1470-2045(20)30641-0 33476593

[10] Jazieh AR Bounedjar A Bamefleh H Alfayea T Almaghraby HQ Belarabi A. Expression of immune response markers in arab patients with lung cancer. JCO Glob Oncol. (2020) 6:1218–24. doi: 10.1200/go.20.00107 PMC7456317 32749860

[11] Al-Shamsi HO Abu-Gheida I Sameh K Tahoun NE Musallam KM. Arab countries and oncology clinical trials: A bibliometric analysis. Cancers (Basel). (2023) 15(18):4428. doi: 10.3390/cancers15184428 37760398 PMC10526906

[12] Khozin S Miksad RA Adami J Boyd M Brown NR Gossai A. Real-world progression, treatment, and survival outcomes during rapid adoption of immunotherapy for advanced non-small cell lung cancer. Cancer. (2019) 125:4019–32. doi: 10.1002/cncr.32383 PMC6899461 31381142

[13] Velcheti V Hu X Yang L Pietanza MC Burke T. Long-term real-world outcomes of first-line pembrolizumab monotherapy for metastatic non-small cell lung cancer with ≥50% Expression of programmed cell death-ligand 1. Front Oncol. (2022) 12:834761. doi: 10.3389/fonc.2022.834761 35402266 PMC8990758

[14] Velcheti V Chandwani S Chen X Pietanza MC Piperdi B Burke T. Outcomes of first-line pembrolizumab monotherapy for PD-L1-positive (TPS ≥50%) metastatic NSCLC at US oncology practices. Immunotherapy. (2019) 11:1541–54. doi: 10.2217/imt-2019-0177 31774363

[15] Ivanović M Knez L Herzog A Kovačević M Cufer T. Immunotherapy for metastatic non-small cell lung cancer: real-world data from an academic central and Eastern European center. Oncologist. (2021) 26:e2143–50. doi: 10.1002/onco.13909 PMC8649015 34288239

[16] Velcheti V Hu X Piperdi B Burke T. Real-world outcomes of first-line pembrolizumab plus pemetrexed-carboplatin for metastatic nonsquamous NSCLC at US oncology practices. Sci Rep. (2021) 11:9222. doi: 10.1038/s41598-021-88453-8 33911121 PMC8080779

[17] Pons-Tostivint E Hulo P Guardiolle V Bodot L Rabeau A Porte M. Real-world multicentre cohort of first-line pembrolizumab alone or in combination with platinum-based chemotherapy in non-small cell lung cancer PD-L1 ≥ 50. Cancer Immunol Immunother. (2023) 72(6):1881–90. doi: 10.1007/s00262-022-03359-2 PMC10198917 36690799

[18] Mok TSK Wu YL Kudaba I Kowalski DM Cho BC Turna HZ. Pembrolizumab versus chemotherapy for previously untreated, PD-L1-expressing, locally advanced or metastatic non-small-cell lung cancer (KEYNOTE-042): a randomised, open-label, controlled, phase 3 trial. Lancet. (2019) 393:1819–30. doi: 10.1016/s0140-6736(18)32409-7 30955977

[19] Waterhouse D Lam J Betts KA Yin L Gao S Yuan Y. Real-world outcomes of immunotherapy-based regimens in first-line advanced non-small cell lung cancer. Lung Cancer (2021) 156:41–9. doi: 10.1016/j.lungcan.2021.04.007 33894493

[20] Liu SV Hu X Li Y Zhao B Burke T Velcheti V. Pembrolizumab-combination therapy for previously untreated metastatic nonsquamous NSCLC: Real-world outcomes at US oncology practices. Front Oncol. (2022) 12:999343. doi: 10.3389/fonc.2022.999343 36324586 PMC9618586

[21] Cramer-van der Welle CM Verschueren MV Tonn M Peters BJM Schramel FMNH Klungel OH. Real-world outcomes versus clinical trial results of immunotherapy in stage IV non-small cell lung cancer (NSCLC) in the Netherlands. Sci Rep. (2021) 11:6306. doi: 10.1038/s41598-021-85696-3 33737641 PMC7973789

[22] Pérol M Felip E Dafni U Polito L Pal N Tsourti Z. Effectiveness of PD-(L)1 inhibitors alone or in combination with platinum-doublet chemotherapy in first-line (1L) non-squamous non-small-cell lung cancer (Nsq-NSCLC) with PD-L1-high expression using real-world data. Ann Oncol. (2022) 33:511–21. doi: 10.1016/j.annonc.2022.02.008 35218887

[23] Ahn BC Pyo KH Xin CF Jung D Shim HS Lee CY. Comprehensive analysis of the characteristics and treatment outcomes of patients with non-small cell lung cancer treated with anti-PD-1 therapy in real-world practice. J Cancer Res Clin Oncol. (2019) 145:1613–23. doi: 10.1007/s00432-019-02899-y PMC6527531 30911841

[24] Descourt R Greillier L Perol M. First-line single-agent pembrolizumab for PD-L1-positive (tumor proportion score >/= 50%) advanced non-small cell lung cancer in the real world: impact in brain metastasis: a national French multicentric cohort (ESCKEYP GFPC study). Cancer Immunol Immunother. (2023) 72:91–9. doi: 10.1007/s00262-022-03232-2 PMC10992810 35729418

[25] Martin C Lupinacci L Perazzo F Bas C Carranza O Puparelli C. Efficacy and safety of nivolumab in previously treated patients with non-small-cell lung cancer: real world experience in Argentina. Clin Lung Cancer. (2020) 21:e380–7. doi: 10.1016/j.cllc.2020.02.014 32213298

[26] Areses Manrique MC Mosquera Martinez J Garcia Gonzalez J Afonso Afonso FJ Lázaro Quintela M Fernández Núñez N. Real world data of nivolumab for previously treated non-small cell lung cancer patients: a Galician lung cancer group clinical experience. Transl Lung Cancer Res. (2018) 7:404–15. doi: 10.21037/tlcr.2018.04.03 PMC6037977 30050778

[27] Morita R Okishio K Shimizu J Saito H Sakai H Kim YH. Real-world effectiveness and safety of nivolumab in patients with non-small cell lung cancer: A multicenter retrospective observational study in Japan. Lung Cancer. (2020) 140:8–18. doi: 10.1016/j.lungcan.2019.11.014 31838169

[28] Dudnik E Moskovitz M Daher S Shamai S Hanovich E Grubstein A. Effectiveness and safety of nivolumab in advanced non-small cell lung cancer: The real-life data. Lung Cancer. (2018) 126:217–23. doi: 10.1016/j.lungcan.2017.11.015 29254746

[29] Park JH You GL Ahn MJ Kim SW Hong MH Han JY. Real-world outcomes of anti-PD1 antibodies in platinum-refractory, PD-L1-positive recurrent and/or metastatic non-small cell lung cancer, and its potential practical predictors: first report from Korean Cancer Study Group LU19-05. J Cancer Res Clin Oncol. (2021) 147:2459–69. doi: 10.1007/s00432-021-03527-4 PMC11802004 33523301

[30] Kobayashi K Nakachi I Naoki K Satomi R Nakamura M Inoue T. Real-world efficacy and safety of nivolumab for advanced non-small-cell lung cancer: A retrospective multicenter analysis. Clin Lung Cancer. (2018) 19:e349–58. doi: 10.1016/j.cllc.2018.01.001 29398578

[31] Nasr L Diab S Ghoche A Yehia I Nasr FL. Real-world outcomes of immunotherapy regimens in first line non small cell lung cancer in lebanese patients. J Clin Oncol. (2022) 40:e18741–1. doi: 10.1200/JCO.2022.40.16_suppl.e18741

[32] El Karak F Gh Haddad F Eid R Al Ghor M El Rassy E Ahmadieh N. Lung cancer and immunotherapy: a real-life experience from second line and beyond. Future Oncol. (2019) 15:3025–32. doi: 10.2217/fon-2019-0144 31424958

[33] Al Nuhait M Bajnaid E Al Otaibi A Al Shammari A Al Awlah Y. Real-world safety experience with immune checkpoint inhibitors in Saudi Arabia. Sci Prog. (2021) 104:36850421997302. doi: 10.1177/0036850421997302 33689534 PMC10358610

[34] Jazieh AR Sağlam EK Önal HC Abdelkader Y Gaafar R Dawoud E. Real-world treatment patterns and outcomes in stage III non-small cell lung cancer: Middle East and Africa - KINDLE study. Clin Lung Cancer. (2022) 23:364–73. doi: 10.1016/j.cllc.2022.02.002 35277345

[35] Petrillo LA El-Jawahri A Nipp RD Lichtenstein MRL Durbin SM Reynolds KL. Performance status and end-of-life care among adults with non-small cell lung cancer receiving immune checkpoint inhibitors. Cancer. (2020) 126:2288–95. doi: 10.1002/cncr.32782 32142165

[36] Friedlaender A Banna GL Buffoni L Addeo A. Poor-performance status assessment of patients with non-small cell lung cancer remains vague and blurred in the immunotherapy era. Curr Oncol Rep. (2019) 21:107. doi: 10.1007/s11912-019-0852-9 31768759

[37] Kano H Ichihara E Harada D Inoue K Kayatani H Hosokawa S. Utility of immune checkpoint inhibitors in non-small-cell lung cancer patients with poor performance status. Cancer Sci. (2020) 111:3739–46. doi: 10.1111/cas.14590 PMC7540975 32726470

[38] Yang B Wang B Chen Y Wan N Xie F Yang N. Effectiveness and safety of pembrolizumab for patients with advanced non-small cell lung cancer in real-world studies and randomized controlled trials: A systematic review and meta-analysis. Front Oncol. (2023) 13:1044327. doi: 10.3389/fonc.2023.1044327 36824127 PMC9942927

[39] Calles A Liao X Sholl LM Rodig SJ Freeman GJ Butaney M. Expression of PD-1 and its ligands, PD-L1 and PD-L2, in smokers and never smokers with KRAS-mutant lung cancer. J Thorac Oncol. (2015) 10:1726–35. doi: 10.1097/jto.0000000000000687 26473645

[40] Herbst RS Baas P Kim DW. Pembrolizumab versus docetaxel for previously treated, PD-L1-positive, advanced non-small-cell lung cancer (KEYNOTE-010): a randomised controlled trial. Lancet. (2016) 387:1540–50. doi: 10.1016/s0140-6736(15)01281-7 26712084

[41] Juergens RA Mariano C Jolivet J Finn N Rothenstein J Reaume MN. Real-world benefit of nivolumab in a Canadian non-small-cell lung cancer cohort. Curr Oncol. (2018) 25:384–92. doi: 10.3747/co.25.4287 PMC6291291 30607113

[42] Mencoboni M Ceppi M Bruzzone M Taveggia P Cavo A Scordamaglia F. Effectiveness and safety of immune checkpoint inhibitors for patients with advanced non small-cell lung cancer in real-world: review and meta-analysis. Cancers (Basel). (2021) 13(6):1388. doi: 10.3390/cancers13061388 33808533 PMC8003199

[43] Castrillon JA Eng C Cheng F. Pharmacogenomics for immunotherapy and immune-related cardiotoxicity. Hum Mol Genet. (2020) 29:R186–r196. doi: 10.1093/hmg/ddaa137 32620943 PMC7574958

[44] Shek D Read SA Ahlenstiel G Piatkov I. Pharmacogenetics of anticancer monoclonal antibodies. Cancer Drug Resist. (2019) 2:69–81. doi: 10.20517/cdr.2018.20 35582142 PMC9019180

